# Look who’s talking now: Cancer in primary care on Twitter. An observational study


**DOI:** 10.3399/bjgpopen20X101134

**Published:** 2021-01-06

**Authors:** Kristi M Milley, Sophie A Chima, Kara-Lynne Cummings, Jon D Emery

**Affiliations:** 1 Primary Care Collaborative Cancer Clinical Trials Group, Centre for Cancer Research and Department of General Practice, Faculty of Medicine, Dentistry and Health Sciences, University of Melbourne, Victorian Comprehensive Cancer Centre, Melbourne, Australia

**Keywords:** primary health care, neoplasms, social media, twitter, general practitioners, cancer

## Abstract

**Background:**

Twitter is a microblogging platform that helps share information. It is a dynamic tool that has been embraced by many user types including consumers and healthcare professionals (HCPs). Currently, there are no data on how cancer in primary care features on Twitter.

**Aim:**

To explore the type of users and information shared about cancer in primary care on Twitter.

**Design & setting:**

A descriptive exploratory study took place of publicly available Twitter data.

**Method:**

Tweets were searched between July 2015 and June 2017 for ‘GP’, ‘general practice’, ‘primary care’, or ‘general practitioner’ in conjunction with ‘cancer’. A 20% random sample was coded for geographic location, user type, type of tweet, and theme. Tweet sentiment was analysed using R package sentimentr. Content that gained traction was compared by combining original tweets, retweets, favourites, and duration.

**Results:**

There were a total of 3413 tweets from 1611 users in 44 countries. Consumers were the largest user group followed by health organisations, healthcare professionals, and the media. The most common theme across user types was diagnostic delay. Other themes that emerged included cancer screening, symptom awareness, and early diagnosis. Consumers published more negative tweets, particularly in relation to diagnostic delay. Health organisations focused on symptom awareness and screening. Over half of media tweets were stories that featured delayed diagnosis or screening.

**Conclusion:**

A broad range of users engage with Twitter to share information about cancer in primary care. Content is different between user groups, but diagnostic delay and symptom awareness are common themes. Healthcare and professional organisations may need to consider approaches to counter negative messages about diagnostic delay.

## How this fits in

Primary care plays an important role in the prevention, early detection, and diagnosis of cancer.^1^ There are also emerging opportunities to further integrate primary care in cancer follow-up and survivorship care.^2,3^ Twitter is a potential platform to communicate this message to different groups of stakeholders, including those at higher risk of cancer. It is an important social media tool that can be used for speed and brevity to communicate with a range of users. This is the first study to explore what content different users share about the role of primary care across the cancer continuum. It provides a foundation to better understand the public perception of primary care in this context. This understanding will allow future research toleverage social media exposure to engage users with content designed to improve awareness of cancer and cancer symptoms, as well as the role of their GP in that journey from prevention through to survivorship.

## Introduction

Social media are powerful communication platforms that are used by over 70% of the general public.^4^ At least half of adult internet users search for or share health-related information.^5^ Increasing knowledge and exchanging advice are two key reasons people engage with social media.^6^ There is great potential for education and communication with the public to disseminate key health messages.^7–9^ The most common social media platforms that feature in health are Facebook and Twitter.^9^ These applications provide a platform for creating^10–13^ and engaging different communities,^14,15^ improving information exchange and communication,^16–23^ and educating a wide range of users.^12,24–28^

Twitter is a microblogging platform that helps capture and share bite-sized pieces of information. Users can share images and video with text up to 280 characters. Launched in 2006, by early 2019 Twitter had grown to nearly 321 million monthly users.^29^ The intersection of Twitter, oncology, education, and communication has been previously studied along with its impact and value for HCPs^18^ including oncologists or haematologists,^21,22,30^ radiologists,^15^ and surgeons.^10^ This has been reviewed in a variety of contexts including scientific conferences,^23,31,32^ networking and education,^33,34^ and improving information access.^10,18,21,35^

In the context of public and patient engagement, there is evidence about the role of Twitter in cancer awareness,^36,37^ developing cancer social networks^16^ and reports of content analysis of cancer-related tweets.^11,27,28,38–42^ Twitter-based interventions have targeted cancer awareness,^26^ patient education, resource access and support,^12^ prevention,^25^ and screening.^14,40^ This evidence is almost exclusively from a secondary or tertiary care perspective, despite the important role primary care and GPs have in the prevention, detection, and early diagnosis of cancer.^43^

As social media becomes increasingly popular it is important to understand how users promote and discuss specific topics. This understanding is an essential foundation to successfully developing targeted campaigns for cancer, which actively engage users and that translate into consumer actions. To date, there is no evidence on how the role of primary care and GPs in cancer are shared and discussed on Twitter. This exploratory study aimed to describe the different types of users and content shared about cancer in primary care, including what type of information generates more user engagement.

## Method

### Search strategy and data collection

Publicly available English language tweets between 30 June 2015 and 30 June 2017 were searched for the terms 'GP' or 'general practitioner' or 'primary care' and 'cancer'. As collection and analysis of these publicly available tweets was an observational study and did not require interaction with any human subjects, it was exempt from institutional review.^44,45^ Tweets were identified, collected, and extracted through a data-services provider, Podargos (Calgary, Canada). A random number generator (Microsoft Excel 365) was used to randomly extract 20% of the tweets collected. Manual coding of the full sample set was not feasible, so a 20% sample size was selected guided by random extraction in existing studies.^36,41,46^

### Data coding

Tweets were assessed for relevance to cancer in primary care. Relevant tweets were then manually coded for the user type, tweet type, and geographic location. The user was determined by reading the biography and most recent tweets of the user account. To compare discussion of the most common cancer types, text was further searched for the terms 'colorectal', 'bowel', 'colon', 'skin', 'melanoma', 'lung', 'prostate', 'prostrate' (sic), 'ovar*', 'cerv*', and 'pancrea*' (the asterisk acts as a wildcard character, to capture within the search results variants that begin with the specified letter string; for example, ’cerv*’ would return **cerv**ix, **cerv**ical, etc). The dataset was coded by one researcher (KM) and the final complete dataset was reviewed by a second researcher (SC) to validate coder reliability. Any conflicts were resolved in consultation with a third team member (JE).

### Data analysis

#### Descriptive analysis

Tweets were provided, coded, and analysed in Microsoft Excel. Descriptive statistics were used to summarise the user type, location, number of tweets per tweet type, and content theme. A quantitative content analysis was used to analyse tweets. A high-order review of tweets followed by an inductive coding approach was used to identify tweet themes. Given the brevity and succinct nature of tweets, each was coded for up to two themes.

#### Sentiment analysis

The overall sentiment of tweets was determined using RStudio (version 1.0.153)^47^ and the package *sentimentr* (version 2.6.1).^48^ A library of around 6800 English words, with predetermined sentiment scores, were used to determine whether the tweets were positive, negative, or neutral.^49,50^ The word ‘cancer’ was removed from the library before being analysed, as the sentiment library classified cancer as a negative word, which would skew analysis. The library also included incorrect spellings of words frequently misspelled on social media. The package scores a tweet between –6 to +6, with –6 being the most negative and +6 being the most positive score.

#### Content engagement

The top stories were determined by reviewing the tweet content and any external links to group together tweets about the same story. The total number of tweets for each story plus the total number of retweets and likes was used to rank the most popular stories. The duration of these stories was determined by calculating the number of active days on Twitter based on the date of the first and last tweet about each story.

## Results

A total of 12 063 tweets were identified. Within the random sample (*n* = 2413), 11.8% (*n* = 284) were not related to cancer in primary care. The remaining 2129 tweets were produced by 1611 unique Twitter accounts (Table 1). Consumers were the largest user group, representing over a quarter of all users. In this context consumers were defined as patients with cancer, their carers, their family members, or members of the general public. Tweets were produced by users from 44 different countries, with the UK, US, Australia, and Canada producing 75% of the content (Table 2).

**Table 1.** Summary of the type of user and frequency of users and tweets

**User type**	**Users, *n* (%**)	**Tweets, *n* (%**)
Consumer	413 (25.6)	457 (21.5)
Health organisation	354 (22.0)	581 (27.3)
Healthcare professional	250 (15.5)	297 (14.0)
Media	229 (14.2)	350 (16.4)
Other	202 (12.5)	258 (12.1)
Unknown	163 (10.2)	186 (8.7)
Total	1611 (100)	2129 (100)

**Table 2.** Summary of tweet origins by geographic location

**Location**	**Tweets, *n***
UK	1078
Unknown	370
US	308
Australia	139
Canada	74
Ireland	33
New Zealand	12
Spain	11
India, Nigeria, South Africa	10
Greece, Pakistan	8
Egypt	6
Belgium, France, Germany	4
Colombia, Indonesia, Japan	3
China, Italy, Nepal, Peru, Saudi Arabia	2
Cyprus, Czech Republic, Ethiopia, Israel, Jamaica, Kenya, Malaysia, Maldives, Malta, Mexico, Namibia, Norway, Oman, Philippines, Russia, Singapore, Sweden, Thailand, Uganda, Zimbabwe	1

Rows with more than one country indicate those countries produced the same number of tweets.

Overall, users’ tweets fitted into one of six categories. These were as follows: sharing news stories about cancer in primary care; promoting results of research studies; promoting a new resource, either for HCPs or consumers; promoting continuing education opportunities; promoting a funding opportunity; or sharing a personal statement.

**Figure 1.**
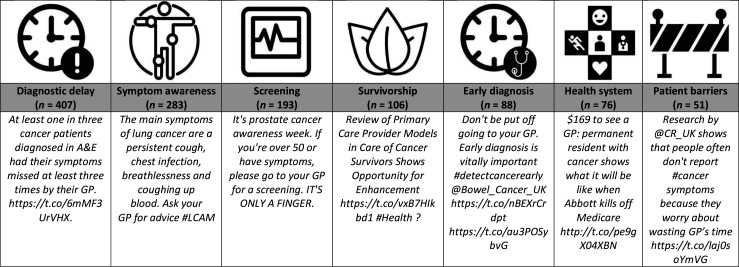
Key themes identified and representative tweets of each theme

Many of the tweets analysed mapped closely to a single aspect of the cancer care continuum. From this, themes that emerged included cancer symptom awareness, prevention, screening, diagnostic delay, survivorship, and palliative care. The most common theme across user types was about a delayed cancer diagnosis (19%). This was followed by symptom awareness (13%), cancer screening (9%), survivorship (5%), and early cancer diagnosis (4%). Other themes were about health system structure (4%) and patient barriers related to investigating possible cancer symptoms (2%) (Figure 1).

**Figure 2.**
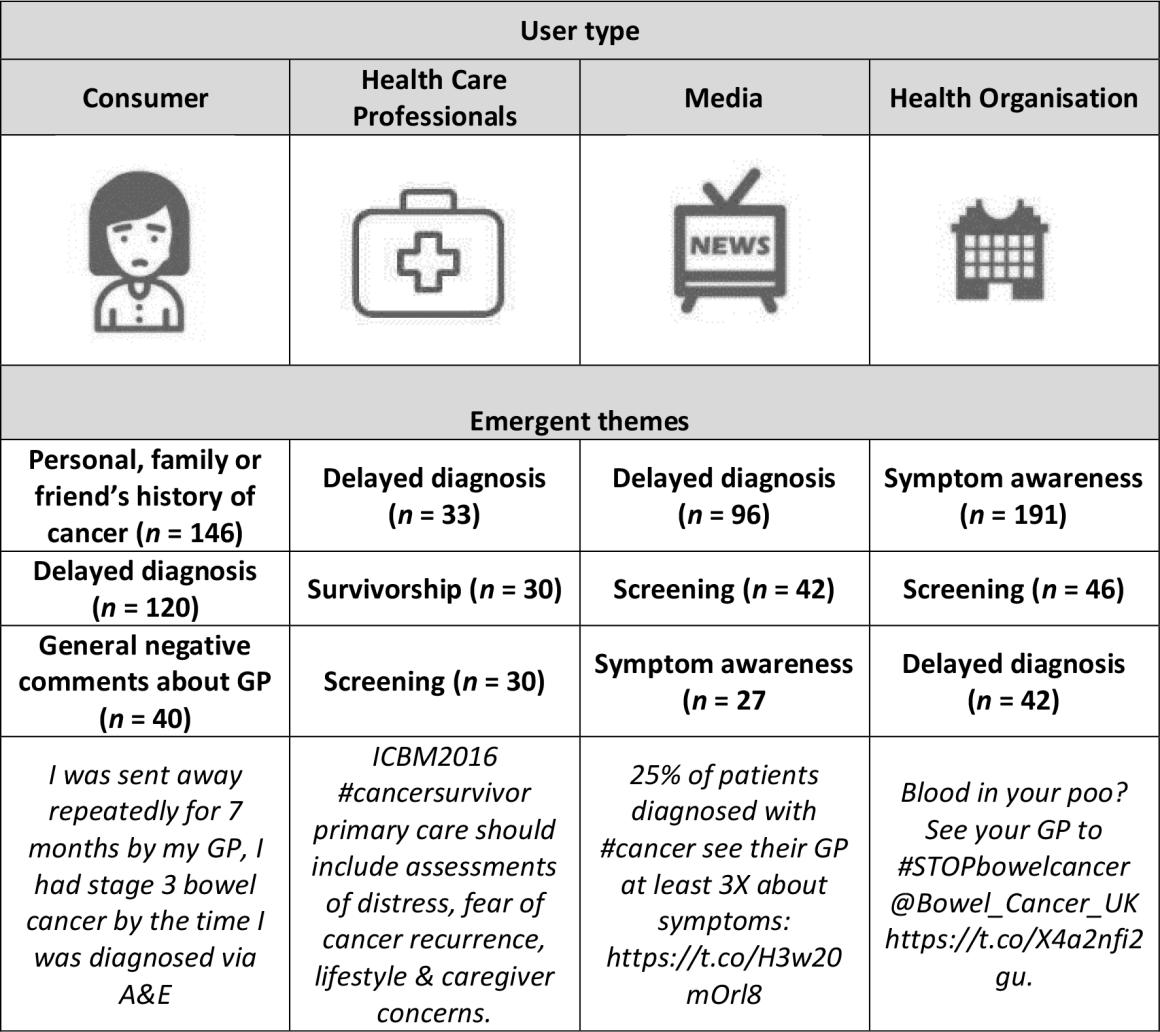
Emergent themes in cancer in primary care tweets by user type: consumers, media, healthcare professionals, and health organisations

Some themes were more common to a specific user type. The most common theme of consumer tweets was a personal comment about their own experience of cancer or that of a friend or family member, in particular about perceived diagnostic delay (Figure 2). This was also a strong theme of media-user tweets (Figure 2). A second theme that featured strongly were stories around cancer screening, often highlighting the importance of primary care in increasing screening participation. HCPs also shared content about diagnostic delay, as well as more content about screening and survivorship (Figure 2). The strongest theme in health organisation tweets was cancer symptom awareness (Figure 2).

A third of tweets contained text about a specific tumour (*n* = 739/2129). Colorectal cancer (33%) was the most common tumour type followed by breast (18%); skin (17%), including both melanoma and non-melanoma skin cancers; lung (11%); and prostate (9%).

Overall, all user groups, except HCPs, produced tweets that were classified as mildly negative with their average score being close to neutral (0). Consumers’ tweets had the most negative scores (Table 3).

**Table 3.** Summary of sentiment analysis scores by user type

	**Sentiment score**	**Average score**
**User type**	-**6**	-**5**	-**4**	-**3**	-**2**	-**1**	**0**	**1**	**2**	**3**	**4**	**5**	**6**	
Consumer	1	2	3	18	44	120	192	57	17	3	0	0	0	–0.42
HCP	0	0	0	7	9	50	131	71	18	9	2	0	0	0.18
Health organisation	0	0	2	16	44	117	278	89	24	9	1	0	0	–0.16
Media	0	0	3	6	26	71	176	59	8	1	0	0	0	–0.21
Other	0	0	0	5	19	46	130	42	6	2	0	0	0	–0.16
Unknown	0	0	5	4	11	35	112	31	7	0	0	0	0	–0.21

HCP = healthcare professional.

**Figure 3.**
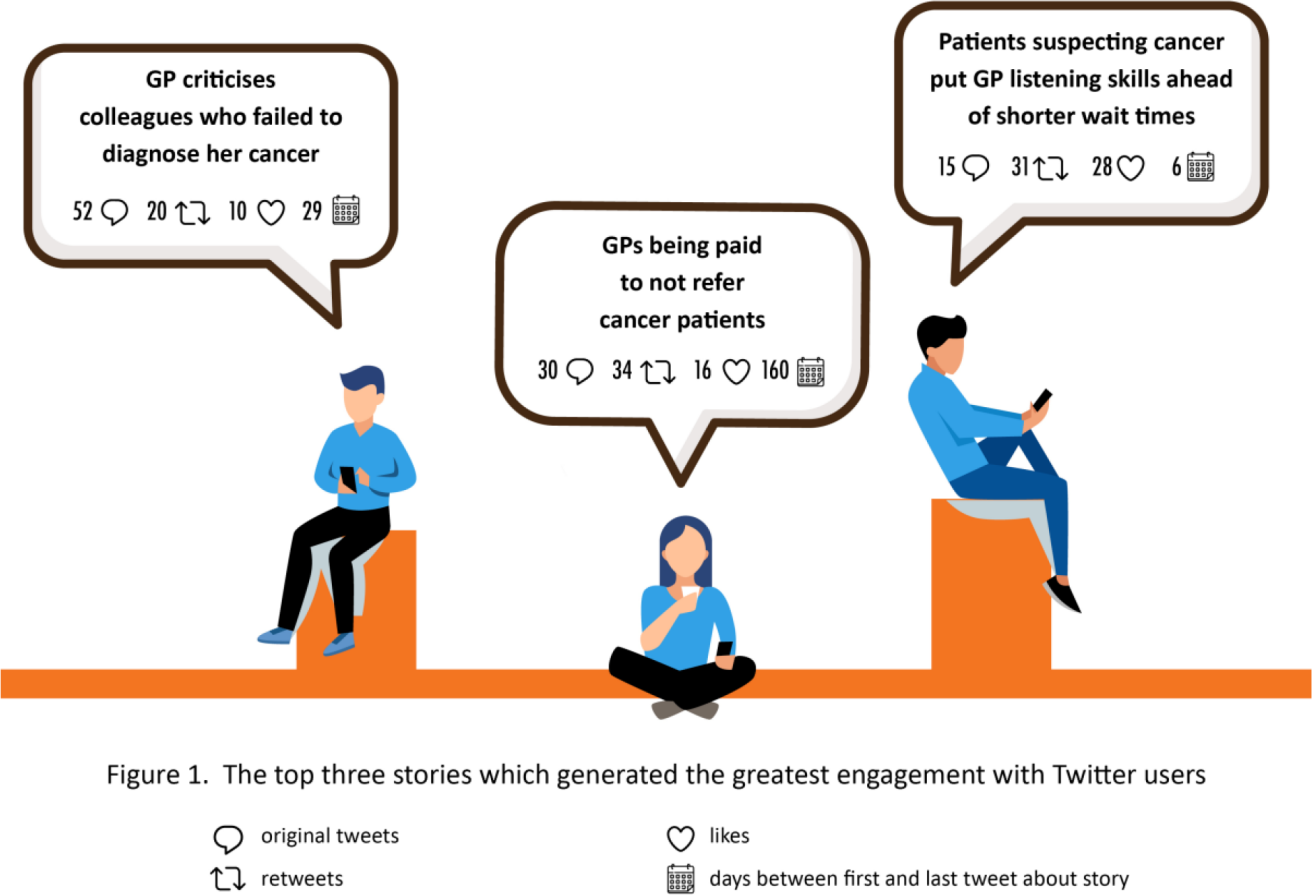
Top three stories that generated the greatest engagement with Twitter users

The top three stories shared by users that produced the greatest engagement were as follows: (1) *'GP criticises colleagues who failed to diagnose her cancer*'; (2) *'GPs being paid not to refer cancer patients*'; and (3) *'Patients suspecting cancer put GP listening skills ahead of shorter wait times*'. These three stories featured on Twitter for between 6 and 160 days (Figure 3).

## Discussion

### Summary

This is the first study to explore the content of tweets about cancer in primary care. Taking into consideration the complexity of a search strategy that could identify all relevant tweets, the number of tweets identified over the 2-year search period highlights that cancer in primary care is a small, niche conversation, among the 500 million tweets sent each day. It is a global conversation but with more content about this subject being contributed by users from the UK. Consequently, this produced many tweets expressed through the lens of lived experience of the UK’s NHS.

Themes of tweets shared by users suggests a negative view around the role of primary care in cancer, particularly during cancer diagnosis. Content shared by both consumers and the media often appeared to perpetuate attitudes and expressions of blame towards GPs. This negative attitude was also clearly evidenced by the tweets that gained the most engagement. These stories highlighted that even though trending on Twitter is often short lived, it does have the capacity to continue to propagate negative content for months.

The negative sentiment expressed by consumers and media agencies warrants further consideration, particularly in the context of shaping the public’s view of the importance of primary care across the cancer continuum. Negative bias presented by the media towards primary care in general has previously been demonstrated.^51^ A review of newspaper articles about neurological illnesses found that over 20% of articles contained medical errors, exaggerations, or stigmatising language.^52^ Primary care nurses have also reported their experiences of the influence of health stories reported in the media, which affected patient perceptions of care and the decisions they made about their care.^53^ In this respect, social media may both perpetuate the problem and be a solution to mediate public perception of cancer in primary care.

The content shared by HCPs suggest that this group use Twitter in a limited capacity. The themes in their tweets suggest they use it as a platform to share educational opportunities and resources with other HCPs. This is in line with other medical disciplines where the focus is on the potential of social media for HCPs in the context of educational and professional opportunities.^18,19,21,22,24,30,33^ The content of HCPs tweets also suggests little communication between GPs and consumers. Instead, the consumer relationship appeared to be nurtured by healthcare organisations, such as cancer-specific charities. Healthcare organisations also appeared to be the only group targeting multiple user groups, where their communications were targeted at HCPs, consumers, and the media.

Quantifying the impact of this conversation on Twitter is difficult, but it is reasonable to consider that a persistent negative perception could have an impact on the views and confidence of the general public in both their personal general practice and primary care more broadly with regards to cancer management, especially early diagnosis. The limited content identified about survivorship highlights there may be an opportunity to increase awareness of the role of primary care and GPs in this space.

### Strengths and limitations

This is the first study to explore the content and themes of tweets shared on Twitter about cancer in primary care. There are limited studies that have undertaken content analysis of tweets and the methodology of these studies is varied. Capturing all involved stakeholders and tweets is difficult given the multitude of potential search terms and combinations to capture relevant tweets.

This study deliberately used the search terms 'general practitioner' and 'GP', which is a common term in Australia and the UK, but may be less commonly used in other countries to describe primary care physicians. The use of the broader ‘primary care’ term could potentially relate to medical professional groups other than GPs or family physicians in the US. There is evidence in other oncology fields that the development of a specific taxonomy and/or hashtags can help focus discussion and identify relevant content.^9^ Promoting primary care-specific hashtags may be a way to better centralise and identify this conversation.

The study presented was performed retrospectively and used available data for a 2-year period up to mid-2017. Consequently, it may not represent the rapidly growing content on Twitter. Additionally, given English language was an inclusion criterion, the analysis of the countries where tweets were posted will be skewed towards English-speaking countries. There are limited data on the validity of using sentiment analysis in this context. Lastly, the analysis did not attempt to identify tweets from ‘bot’ accounts. Up to 15% of Twitter accounts may be automated ‘bot’ accounts.^54^ The study was unable to estimate the impact of these accounts on propagating tweets or specific tweet themes. The reasoning was that this study was about what content was shared and engaged with by users. As such, there was no impact on the analysis if the content was produced by an automated account or a live person.

### Comparison with existing literature

This is the first study to explore the content of conversations on Twitter about cancer in primary care. A 2017 systematic review of Twitter as a tool for health research found that only 5% of included studies were focused on cancer. None of the studies identified explicitly involved primary care. The most common fields were public health, infectious disease, behavioural medicine, and psychiatry.^55^

### Implications for research and practice

This study raises a question about if there is a need for primary care organisations to counter negative opinions and content around the role of primary care in cancer presented on Twitter. There is limited data on the impact of information shared by users on Twitter. In the age of influencers, understanding the spread and impact of content may help inform the development of targeted messages or awareness campaigns. It may also help identify primary care champions, users that are successfully supporting the role of primary care in cancer. In other disciplines, tweet chats have been used to engage in discussions with patients, although this has been limited to patients with breast and lung cancer.^16,56^

Cancer in primary care is a niche conversation on Twitter but one in which the media and consumers, and their cancer experiences feature prominently. Predominatly, these experiences were negative. There was a distinct absence of substantial positive promotion of primary care. Themes that emerged centred around perceived diagnostic delay, screening, and symptom awareness. As the role of primary care in cancer survivorship continues to grow there is an opportunity to increase awareness through Twitter and other social media platforms. Future research should explore the impact of negative sentiment on public perceptions of primary care and how organisations could influence sentiment within this platform to promote the importance of high-quality primary care across the cancer continuum.
